# Laser Diode Pumped Polymer Lasers with Tunable Emission Based on Microfluidic Channels

**DOI:** 10.3390/polym13203511

**Published:** 2021-10-13

**Authors:** Ben Niu, Kun Ge, Zhiyang Xu, Xiaoyu Shi, Dan Guo, Tianrui Zhai

**Affiliations:** Faculty of Science, College of Physics and Optoelectronics, Beijing University of Technology, Beijing 100124, China; niubenlaser@163.com (B.N.); GEKUN@emails.bjut.edu.cn (K.G.); xu.zhiyang@hotmail.com (Z.X.); xyshi@bjut.edu.cn (X.S.); dguo@bjut.edu.cn (D.G.)

**Keywords:** tunable laser, laser diode pumped, polymer, microfluidic channel

## Abstract

Tunable whispering-gallery-mode (WGM) lasers have been paid lots of attention for their potential applications in the photonic field. Here, a tunable polymer WGM laser based on laser diode pumping is realized with a threshold of 0.43 MW/cm^2^ per pulse. The WGM laser is realized by a microfluidic microcavity, which consists of a quartz capillary and gain materials. The laser performance keeps stable for a long time (3.5 h), pumped by a 50-ns 50 Hz laser diode with a pumping peak power density of 1.08 MW/cm^2^ per pulse. The lasing wavelength can be tuned over 15 nm by changing the gain material concentration from 3.5 mg/mL to 12.5 mg/mL in the microfluidic channel. Moreover, the lasing mode can be switched between transverse magnetic (TM) and transverse electric (TE) modes by adjusting the pump polarization. These results provide the basis for designing nanophotonic devices with laser diode pumping.

## 1. Introduction

In recent years, polymer lasers have attracted more and more attention because of the simple fabricating, broad absorption and emission spectra, large tuning range, high photoluminescence quantum yield and large absorption cross-section [1,2,3,4,5,6,7]. They are widely used in spectroscopy [8], bio/chem-sensing [9,10], full color laser display [11,12,13] and information processing [14]. However, those polymer lasers are generally optically pumped by complex femtosecond lasers or frequency doubled/tripled solid state lasers [15,16,17,18], which are complex and much more expensive than the polymer laser itself, greatly hindering the application of those lasers. At the same time, electrical pumping is still an open challenge [19]. With the rapid development of blue-violet emitting inorganic laser diodes and polymer material, an increasing number of polymer semiconductor lasers pumped by laser diodes or light emitting diodes have been demonstrated [20,21,22,23], paving the way for compact and truly low-cost polymer semiconductor laser systems, and greatly promoting their applications.

Nevertheless, the peak power of laser diodes is weak compared to the values achieved with pulsed femtosecond lasers, the threshold of polymer laser resonators should be as low as possible. Recently, diode laser pumped polymer lasers have been achieved up to now through the use of a few resonators, such as distributed feedback (DFB) [24,25,26], vertical cavity surface emitting polymer laser (VCSEL) resonators [27], and astigmatism-compensated three-mirror cavity [28,29]. The WGM microcavity lasers are widely used for sensing because of their high Q factor and low threshold [30,31,32,33]. However, the laser diode pumped polymer WGM lasers have been rarely reported up to now.

In this paper, we realize a wavelength tunable WGM laser pumped by a pulsed commercial blue laser diode, and the WGM microcavity consisting of the quartz capillary. The quartz capillary serves as a microfluidic channel to achieve multiple lasing modes. The WGM lasing has a low threshold of 0.43 MW/cm^2^ per pulse and the high quality (Q) factor is close to 10^4^. The device has excellent photostability, therefore the output emission density is not significantly weakened after 3.5 h pumping with a linewidth of 50 ns and repetition frequency of 50 Hz when the pumping peak power density is 1.08 MW/cm^2^ per pulse. The peak wavelength of the microfluidic channel microcavity red-shifts over 15 nm by altering the dye concentration from 3.5 mg/mL to 12.5 mg/mL. At the same time, the emission intensity of the WGM lasing can be controlled by changing the pump polarization characteristics. Our work will provide the basis for designing nanophotonic devices with diode pumping.

## 2. Materials and Methods

**Figure 1:**
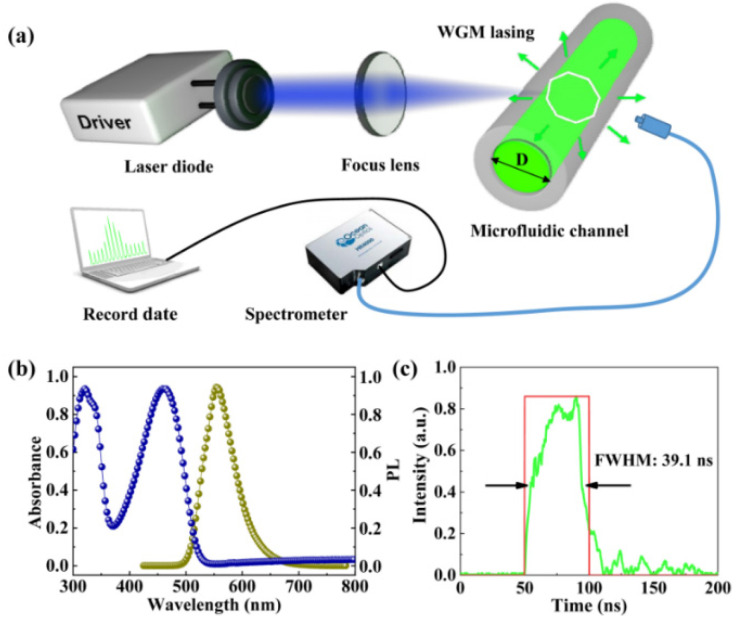
(**a**) Absorption (blue line) and photoluminescence (brown line) spectra of F8BT, with its absorption peak around 450 nm. (**b**) The schematic diagram of laser diode pumped polymer WGM lasers. (**c**) The actual full width at half maxima (FWHM) of the diode emission pulse is only 39.1 ns when the driving pulse is 50 ns.

The typical light-emitting polymer poly[(9,9-dioctylfluorenyl-2,7-diyl)-alt-co-(1,4-benzo-(2,1’,3) thiadiazole)] (F8BT, American Dye Source) is employed as an active material and dissolved in xylene. The absorption and photoluminescence (PL) characteristics of F8BT are shown in Figure 1b. The F8BT solution is wicked into a commercial quartz capillary (Zhong Cheng Quartz Glass Co., Ltd. Beijing, China) microcavity with an inner diameter of 0.3 mm and wall thickness of 0.15 mm, respectively, forming a green light-emitting polymer WGM laser resonator. The schematic of the investigated laser diode pumped polymer WGM laser is shown in Figure 1a.

A commercial blue inorganic laser diode (NDB7Y75, Nichia Corporation) with two aspheric lens is used as the pump source. The laser diode with the maximum continuous output power of 5 W and the central emission wavelength of 450 nm was driven by a pulsed power supply (Picolas GmbH). The threshold current is 0.32 A and slope efficiency is 1.8 W/A. The astigmatic and highly divergent output beam is collimated and focused to an elliptical spot size with a diameter along the fast and slow axis of 43 μm and 116 μm, respectively. The advantage of the laser diode acting as pumping source is not only low-cost but also offers a totally user-controlled pulse duration, and the pulse duration can be altered from a few nanoseconds to a continuous wave (CW). Because the drive pulse takes time to rise and fall, when the electrically driven pulse is set to 50 ns, the actual width (full width at half maximum, FWHM) of the diode emission pulse is only 39.1 ns, as shown in Figure 1c. The laser diode, cooled by natural convection, can be driven up to 40 A with the pulse duration of 50 ns and the repetition frequency of 50 Hz. The corresponding peak power density is 1.44 MW/cm^2^ per pulse. A spectrometer (HR 4000, Ocean Optics) with a spectral resolution of 0.01 nm is used to collect the emission of the WGM lasers.

## 3. Results

**Figure 2:**
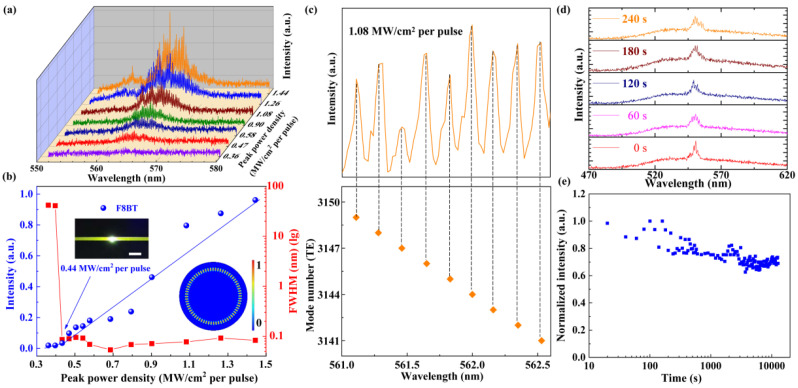
(**a**) The emission spectrum of the diode pumped tunable polymer WMG laser. (**b**) The intensity and the FWHM of the output emission at a different current, demonstrating the lasing threshold is 0.43 MW/cm^2^ per pulse marked by an arrow. Top left inset is the optical image of microfluidic channel. Scale bar is 800 μm. Bottom right illustration is the distribution of electric field intensity in transverse cross-section. (**c**) The lasing spectra from the quartz capillary, mλ_m_ = πn_eff_D, where m is mode number, λ_m_ is the wavelength. The corresponding mode number is from 3141 to 3149. (**d**) The evolution of emission spectrum from 0 s to 240 s. (**e**) The stability of laser diode pumped polymer WGM laser.

In the experiment, the quartz capillary is chosen as a WGM microcavity as the surface of the capillary is smooth and uniform, which can serve as a microfluidic channel to achieve multiple lasing modes. The evolution of PL spectrum of the laser diode pumped WGM laser is demonstrated when the pumping peak power density ranges from 0.36 MW/cm^2^ per pulse to 1.44 MW/cm^2^ per pulse, as shown in Figure 2a. Only a broad spontaneous emission spectrum peak at 565 nm is observed when the pumping peak power density is lower than 0.43 MW/cm^2^ per pulse, and the FWHM is over 30 nm. Moreover, several equidistant narrow laser peaks are clearly demonstrated with the pumping peak power density exceeding 0.43 MW/cm^2^ per pulse, the intensity of the emission spectrum is increased and FWHM of emission spectrum is narrowed quickly lower than 0.1 nm. These features indicate that the resonant feedback is built up in the microfluidic channel. Figure 2b shows the relationship between the normalized density and the FWHM of output emission spectrum and different current, demonstrating that the threshold is about 0.43 MW/cm^2^ per pulse marked by an arrow. The top left inset is the optical image of the microfluidic channel. The scale bar is 800 μm. The bottom right illustration is the distribution of simulated electric field intensity in the transverse cross-section, as shown in Figure 2b. The electric field intensity distribution in the transverse cross-section is numerically simulated with the commercial software COMSOL multi-physics 5.4. The radius is set to 3 μm in the simulation in order to obtain a clear and visible mode field distribution. The numerically simulated results show that the quartz capillary can support the WGM lasing resonance, and the modes distribute near the inner surface of microfluidic channel. The simulation parameters include the emission center wavelength of 550 nm and the effective refractive index of 1.55. All parameters are derived from the experimental measurements.

The theoretical calculation demonstrates the lasing peaks that belong to the first order TE modes, and the corresponding mode number is from 3141 to 3149 as shown in Figure 2c. In the experiment, we keep the current 1.08 MW/cm^2^ per pulse and record the PL spectrum when the WGM microcavity is stimulated by the laser diode in the microfluidic channel. According to the WGM theory

mλ_m_ = πn_eff_D (1)

where λ_m_ is peak wavelength and n_eff_ is effective refractive index of the gain solution, and D is the inner diameter of the microfluidic channel, respectively. The photostability of this device is investigated at fixed pumping peak power density of 1.08 MW/cm^2^ per pulse with a pulse width of 50 ns and repetition frequency of 50 Hz. the output emission density is not significantly weakened after being pumped after 3.5 h. The evolution of the PL emission spectrum is recorded from 0 s to 240 s, as shown in Figure 2d, and the intensity variation in 12,700 s is shown in Figure 2e. The refractive index of polymer solution is calculated and fitted by the spectroscopic ellipsometer (ESNano, ELLiTOP Sclentiflo Co., Ltd. Beijing, China).

**Figure 3:**
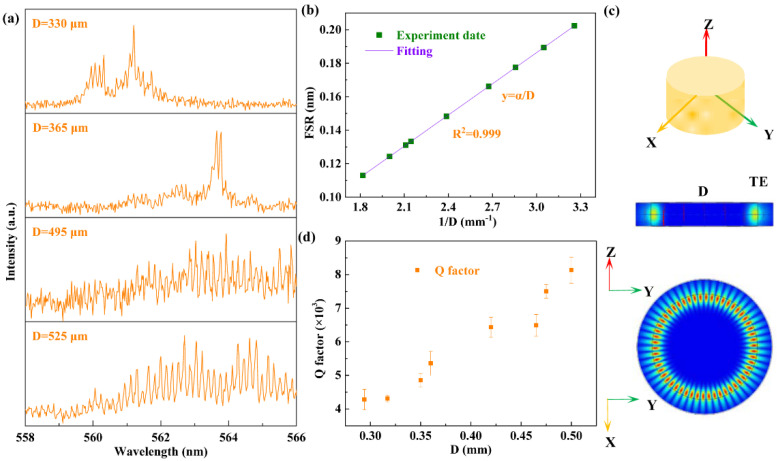
(**a**) The WGM modulation in four microfluidic channels with different diameters above the threshold. (**b**) The relationship between FSR and 1/D, the solid line is fitted with the Equation (2). (**c**) The structure diagram of the microfluidic channel, the cross section of electric field distribution of TE mode. (**d**) The relationship between Q factor and various diameters.

The mode spacing and Q factor can be tuned by changing the size of microcavity. The mode spacing gradually increases with the decreased dimensions from top to bottom as shown in Figure 3a. The FSR are 0.125 nm, 0.132 nm, 0.179 nm and 0.198 nm with the microcavities of 525 μm, 495 μm, 365 μm and 330 μm, respectively, as shown in the Figure 3a. Figure 3b shows the relationship between FSR and 1/D, the corresponding effective refractive index is 1.55. Hence, the FSR for the WGM laser can be illustrated as:

(2) $$\mathrm{FSR}=\frac{\lambda^{2}}{{\pi n}_{\mathrm{eff}}D}$$

where λ is peak wavelength and neff is effective refractive index of the active material, and D is the inner diameter of the inner microfluidic channel, respectively. The solid line is fitted with the Equation (2). The slope α is constant, which shows the linear relationship between free spectral range (FSR) and the diameter of quartz capillary, as shown in Figure 3b. Moreover, the calculation of Q factor is in the range from 4000 to 8000, the triangles represent the relationship between Q factor and various diameters in the experiment in Figure 3d. In our experiment, the Q value can be estimated as 10^3^ by using the equation Q = λ⁄Δλ, where λ is 565 nm and Δλ is 0.11 nm.

The electric field intensity distribution of TE mode in the transverse cross-section is numerically simulated with the commercial software COMSOL multi-physics 5.4. The numerically simulated results show that the quartz capillary can support the WGM lasing resonance and oscillation in the surface of microfluidic channel. The scaling law is used in the simulation. The radius is scaled to 0.9μm and the center wavelength is scaled to 250 nm. The simulation parameters, including the inner and outer diameters of the quartz capillary, are 0.7 μm and 0.9 μm, respectively. The effective refractive index is 1.55. The simulating results are shown in Figure 3c.

**Figure 4:**
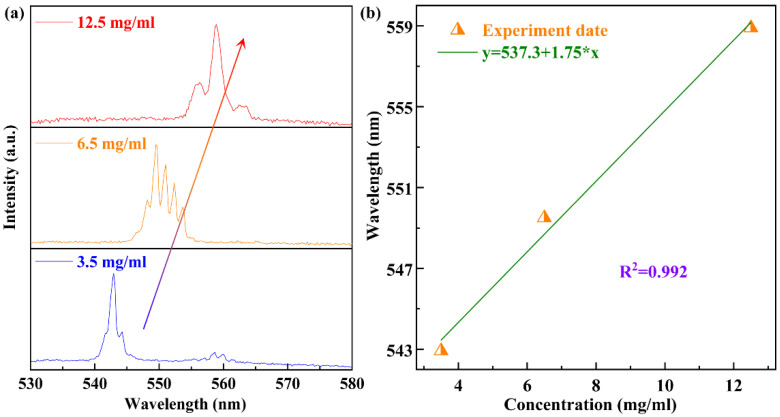
(**a**) The evolution of the WGM lasing of spectrum with different gain concentration from 3.5 mg/mL to 12.5 mg/mL. (**b**) The relationship between gain concentration and emission wavelength.

The wavelength of the diode pumped polymer WGM laser can be tuned by altering the dye concentration. The wavelength is observed red-shifting, with increasing concentration of active material due to increasing absorption as shown in Figure 4. Similarly, the wavelength is blue-shifting with decreasing concentration of active material due to decreasing absorption. According to the theory, we have achieved the diode pumped tunable polymer WGM lasing, and the wavelength can be tuned over 15 nm by change the dye concentration from 3.5 mg/mL to 12.5 mg/mL.

**Figure 5:**
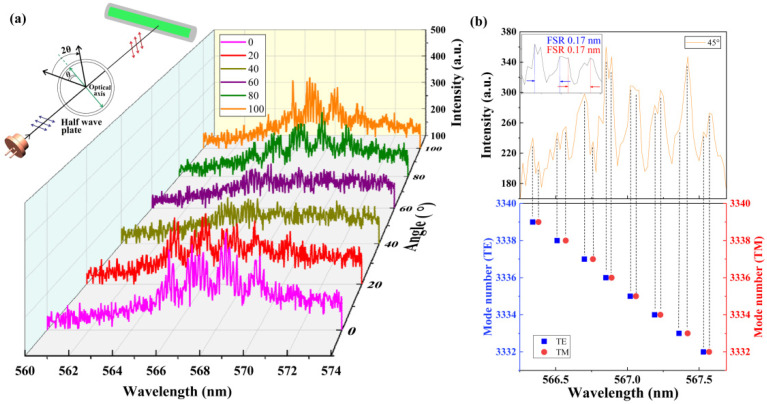
(**a**) The emission spectrum intensity changes periodically with the change of pump polarization, caused by the switch of the output mode between TE and TM mode. Inset is the schematic diagram of mode switching. (**b**) The spectrum of mode switching critical point.

The relationship between the laser output mode and the polarization characteristics of the pump is also investigated. A rotatable half wave plate is placed in front of the laser diode to change the polarization direction of the pump. The emission intensity of the WGM lasing can be changed by rotating the half wave plate. The evolution of spectrum under different angles from 0° to 100° is shown in Figure 5a. We also found that the pump polarization characteristics can switch the output mode between TE mode and TM mode. Inset is the schematic diagram of mode switching (in Figure 5a). Figure 5b provides the spectrum of mode switching between TE mode and TM mode. The FSR does not change when mode switching is between TE mode and TM mode with rotation angle of 45°, as shown in the inset of Figure 5b.

## 4. Conclusions

In summary, we have realized a wavelength tunable WGM laser by laser diode pumping in a microfluidic channel with a low threshold of 0.43 MW/cm^2^ per pulse. The device is photostable and the output lasing density is not significantly weakened after 3.5 h with the pumping peak power density of 1.08 MW/cm^2^ per pulse. The pulse width is 50 ns and the repetition frequency is 50 Hz. The lasing mode of the diode pumped polymer WGM laser red-shifts over 15 nm when altering the dye concentration from 3.5 mg/mL to 12.5 mg/mL. Moreover, the pump polarization characteristics can switch the output mode between TE mode and TM mode. Our work will provide the basis for designing nanophotonic devices with laser diode pumping.

## Data Availability

The data presented in this study are available on request from the corresponding author.

## References

[1] Muller C.D., Falcou A., Reckefuss N.M., Wiederhirn V., Rudati P., Frohne H., Nuyken O., Becker H., Meerholz K. (2003). Multi-colour polymer light-emitting displays by solution processing. Nature.

[2] Kim T.H., Cho K.S., Lee E.K., Lee S.J., Chae J., Kim J.W., Kim D.H., Kwon J.Y., Amaratunga G.A., Lee S.Y. (2011). Full-colour quantum dot displays fabricated by transfer printing. Nat. Photon..

[3] Fu Y.L., Zhai T.R. (2020). Distributed feedback polymer lasing in photonic crystals. Front. Optoelectron..

[4] Dang C., Lee J., Breen C., Steckel J.S., Coe-Sullivan S., Nurmikko A. (2012). Red, green and blue lasing enabled by single-exciton gain in colloidal quantum dot films. Nat. Nanotechnol..

[5] Zhai T.R., Xu Z.Y., Li S.T., Zhang X.P. (2017). Red-green-blue plasmonic random laser. Opt. Express.

[6] Du Y.X., Zou C.L., Zhang C.H., Wang K., Qiao C., Yao J.N., Zhao Y.S. (2020). Tunable red, green, and blue single-mode lasing in heterogeneously coupled polymer spherical microcavities. Light Sci. Appl..

[7] Li S.T., Wang L., Zhai T.R., Niu L.Z., Tong F., Cao F.Z., Wang M., Zhang X.P. (2018). Red-green-blue plasmonic random lasing from cascaded polymer slices. Laser Phys. Lett..

[8] Liu X., Stefanou P., Wang B.H., Woggon T., Mappes T., Lemmer U. (2013). Polymer semiconductor distributed feedback (DFB) laser as excitation source in Raman spectroscopy. Opt. Express.

[9] Wang Y., Morawska P.O., Kanibolotsky A.L., Skabara P.J., Turnbull G.A., Samuel I.D.W. (2013). LED pumped polymer laser sensor for explosives. Laser Photonics Rev..

[10] Rose A., Zhu Z.G., Madigan C.F., Swager T.M., Bulovic V. (2005). Sensitivity gains in chemosensing by lasing action in polymer polymers. Nature.

[11] Ge K., Guo D., Ma X.J., Xu Z.Y., Hayat A., Li S.T., Zhai T.R. (2021). Large-area biocompatible random laser for wearable applications. Nanomaterials.

[12] Chen M.J., Hu S.Q., Zhou Z.W., Huang N., Lee S., Zhang Y.G., Cheng R., Yang J., Xu Z.Y., Liu Y. (2021). Three-Dimensional Perovskite Nanopixels for Ultrahigh-Resolution Color Displays and Multilevel Anticounterfeiting. Nano Lett..

[13] Yin Y.M., Hu Z.P., Ali M.U., Duan M., Gao L., Liu M., Peng W.X., Geng J., Pan S., Wu Y.W. (2020). Full-color micro-LED display with CsPbBr3 perovskite and CdSe quantum dots as color conversion layers. Adv. Mater. Technol..

[14] Ma B.W., Zou W.W. (2020). Demonstration of a distributed feedback laser diode working as a graded-potential signaling photonic neuron and its application to neuromorphic information processing. Sci. China Inf. Sci..

[15] Samuel I.D.W., Turnbull G.A. (2007). Polymer semiconductor lasers. Chem. Rev..

[16] Tsutsumia N., Fujihara A. (2005). Tunable distributed feedback lasing with narrowed emission using holographic dynamic gratings in a polymeric waveguide. Appl. Phys. Lett..

[17] Hayat A., Tong J.H., Chen C., Niu L.Z., Aziz G., Zhai T.R., Zhang X.P. (2020). Multi-wavelength colloidal quantum dot lasers in distributed feedback cavities. Sci. China Inf. Sci..

[18] Haghighi H.R., Forget S., Chénais S., Siove A. (2010). Highly efficient, diffraction-limited laser emission from a vertical external-cavity surface-emitting polymer laser. Opt. Lett..

[19] Zhang Q., Zeng W.J., Xia R.D. (2015). Current reflearch and future development of polymer laser materials and devices. Acta Phys. Sin..

[20] Vasdekis A.E., Tsiminis G., Ribierre J.C., Faolain L.O., Krauss T.F., Turnbull G.A., Samuel I.D.W. (2006). Diode pumped distributed Bragg reflector lasers based on a dye-to-polymer energy transfer blend. Opt. Express.

[21] Karnutsch C., Stroisch M., Punke M., Lemmer U., Wang J., Weimann T. (2007). Laser diode-pumped polymer semiconductor lasers utilizing two-dimensional photonic crystal resonators. IEEE Photon. Technol. Lett..

[22] Sakataa H., Takeuchi H. (2008). Diode-pumped polymeric dye lasers operating at a pump power level of 10 mW. Appl. Phys. Lett..

[23] Stefanska D., Suski M., Zygmunt A., Stachera J., Furmann B. (2019). Tunable single-mode CW energy-transfer dye laser directly optically pumped by a diode laser. Opt. Laser Technol..

[24] Klinkhammer S., Liu X., Huska K., Shen Y.X., Vanderheiden S., Valouch S., Vannahme C., Brase S., Mappes T., Lemmer U. (2012). Continuously tunable solution-processed polymer semiconductor DFB lasers pumped by laser diode. Opt. Express.

[25] Tsiminis G., Wang Y., Kanibolotsky A.L., Inigo A.R., Skabara P.J., Samuel I.D.W., Turnbull G.A. (2013). Nanoimprinted polymer semiconductor laser pumped by a light-emitting diode. Adv. Mater..

[26] Foucher C., Guilhabert B., Herrnsdorf J., Laurand N., Dawson M.D. (2014). Diode-pumped, mechanically-flexible polymer DFB laser encapsulated by glass membranes. Opt. Express.

[27] Zhao Z., Mhibik O., Nafa M., Chenais S., Forget S. (2015). High brightness diode-pumped polymer solid-state laser. Appl. Phys. Lett..

[28] Burdukova1 O.A., Gorbunkov M.V., Petukhov V.A., Semenov M.A. (2016). Diode pumped dye laser. Laser Phys. Lett..

[29] Burdukova1 O., Gorbunkov M., Petukhov V., Semenov M. (2017). Diode pumped tunable dye laser. Appl. Phys. B.

[30] Andreas L., Regine F. (2020). Quantum Many-Body Theory for Exciton-Polaritons in Semiconductor Mie Resonators in the Non-Equilibrium. Appl. Sci..

[31] Andreas L., Regine F. (2019). A Self-Consistent Quantum Field Theory for Random Lasing. Appl. Sci..

[32] Dai J., Xu C.X., Sun X.W., Zhang H.X. (2011). Exciton-polariton microphotoluminescence and lasing from ZnO whispering-gallery mode microcavities. Appl. Phys. Lett..

[33] Duan Q.Q., Xu D., Liu W.H., Lu J., Zhang L., Wang J., Wang Y.L., Gu J., Hu T., Xie W. (2013). Polariton lasing of quasi-whispering gallery modes in a ZnO microwire. Appl. Phys. Lett..

